# Explainable machine learning for osteoporosis detection in patients with osteopenia: model development and validation using routine clinical data from an Asian cohort

**DOI:** 10.3389/fendo.2026.1857227

**Published:** 2026-07-20

**Authors:** Xiuzhen Zhang, Li Zhao, Han Wu, Fengyi Yuan, Weiqing Wu, Yan Wu, Wei Wang

**Affiliations:** 1 Department of Endocrinology and Metabolism, Shenzhen People’s Hospital (The First Affiliated Hospital, Southern University of Science and Technology; The Second Clinical Medical College, Jinan University), Shenzhen, Guangdong, China; 2 Health Management Center, Shenzhen People’s Hospital (The First Affiliated Hospital, Southern University of Science and Technology; The Second Clinical Medical College, Jinan University), Shenzhen, Guangdong, China

**Keywords:** DXA-independent, machine learning, osteopenia, osteoporosis, risk stratification

## Abstract

**Background:**

Osteopenia is a critical precursor to osteoporosis (OP), yet accurately discriminating OP from osteopenia among individuals with low bone mass remains challenging. While dual-energy X-ray absorptiometry (DXA) provides definitive diagnosis, accessibility limitations necessitate alternative screening approaches. We therefore aimed to develop an algorithm based on readily available clinical data to discriminate between OP and osteopenia in this population.

**Methods:**

We conducted a retrospective diagnostic study to develop a model for discriminating osteoporosis from osteopenia within a cohort of 1,203 Asian adults with low bone mass. Eleven machine learning algorithms were trained and validated for this diagnostic task (case: osteoporosis [T-score ≤ -2.5]; control: osteopenia [T-score -2.5 to -1.0]).Performance was evaluated using area under the curve (AUC). The interpretability and clinical utility of the selected model were respectively enhanced and validated by SHAP analysis, nomogram calibration, and decision curve analysis (DCA).

**Findings:**

The Linear Discriminant Analysis model demonstrated superior and consistent performance. It achieved a mean cross-validated AUC of 0.738 (95% CI: 0.736–0.741) and showed excellent calibration (Hosmer-Lemeshow p = 0.266). On an independent validation set, the model maintained robust performance with an AUC of 0.710 (95% CI: 0.686–0.734). Key predictors included waist-to-height ratio, body weight, serum uric acid, age, and alkaline phosphatase. DCA indicated a positive net benefit across a wide range of risk thresholds.

**Interpretation:**

This study developed a practical and interpretable tool for discriminating osteoporosis from osteopenia among individuals with low bone mass, using only routinely available clinical data. This approach may serve as a preliminary screening tool to identify high-risk individuals within primary care populations for further definitive testing.

## Introduction

Osteoporosis (OP) constitutes a growing public health crisis in Asia, with epidemiological studies projecting that the region will account for more than half of all osteoporotic fractures worldwide by 2050 (1, 2). This burden is intensified by substantial disparities in diagnostic resources. In many Western health systems, population-wide dual-energy X-ray absorptiometry (DXA) screening programs have achieved coverage rates exceeding 60% among high-risk groups (3, 4). In contrast, access to DXA remains notably limited across much of Asia, particularly in rural areas, where availability drops by approximately 25% and services are often unavailable altogether (5, 6). As a result, a significant diagnostic gap exists, disproportionately affecting the estimated 40% of middle-aged and older Asian adults living with osteopenia (7, 8), a population at high risk of OP yet largely overlooked by current screening frameworks.

Current risk-assessment approaches face several limitations. First, clinical guidelines offer few evidence-based strategies for managing osteopenia in resource-limited settings. Second, machine learning (ML) models for OP prediction have been predominantly developed using Western populations (8, 9), despite known ethnic differences in bone mineral density (BMD) patterns. Third, many existing algorithms rely on specialized biomarkers or advanced imaging data (10–13) that are seldom available in routine primary care, which serve as first point of contact for over 80% of the Asian population.

To bridge these gaps, we conducted a retrospective study with three key objectives: (1) to develop and validate a diagnostic model specifically for discriminating osteoporosis from osteopenia within an Asian cohort with confirmed low bone mass, enhancing population relevance; (2) to employ only routine clinical variables obtainable in primary care settings; and (3) to integrate explainable artificial intelligence (AI) methods to improve model interpretability. A distinctive aspect of our approach is the deliberate exclusion of DXA-based parameters during model development, aiming to create a practical screening tool that does not depend on advanced diagnostic infrastructure. By systematically comparing eleven ML algorithms, we seek to identify a model that balances predictive performance with clinical applicability for cross-sectional discrimination between OP and osteopenia in low bone mass individuals.

## 2 Materials and methods

### 2.1 Study cohort

This single-center retrospective cohort study analyzed routinely collected health examination data from the Health Management Center of Shenzhen People’s Hospital, covering the period from January 2019 to December 2024. Inclusion criteria: (1) Adults aged ≥18 years who underwent BMD assessment via DXA using a Hologic Discovery A scanner (scanning precision: ± 0.01 g/cm²), with valid measurements at three anatomical sites (lumbar spine L2-L4, femoral neck, total hip); (2) Complete clinical datasets, including anthropometric parameters and laboratory results, with ≤20% missing key data. Key variables were defined as demographic characteristics, metabolic indicators, inflammatory markers, and calculated metabolic-inflammatory indices (detailed in **Supplementary Table 2**). Exclusion criteria: (1)Age < 18 years; (2)Secondary bone diseases (e.g., rheumatoid arthritis, hyperparathyroidism, osteogenesis imperfecta, chronic kidney disease-related osteodystrophy);(3)Use of OP-targeted medications within 1 year (e.g., bisphosphonates, selective estrogen receptor modulators, denosumab, teriparatide);(4)Use of bone metabolism-affecting drugs within 6 months (including but not limited to glucocorticoids, thyroid hormones, antiepileptic drugs, and proton pump inhibitors);(5)Active malignancy;(6)Diabetes mellitus (type 1 or type 2, confirmed by medical records or glycated hemoglobin ≥6.5%);(7)History of spinal/hip surgery or fracture within 1 year;(8)Pregnant or lactating women; (9)Missing key data > 20%. Final analytic cohort refinement: To align with the study’s core objective of discriminating OP from osteopenia in individuals with impaired bone health, participants with normal BMD were excluded. This refinement was justified by two key considerations: (1) clinical relevance—individuals with normal BMD do not require urgent priority intervention, whereas the osteopenia subgroup represents the critical target population for early risk stratification; (2) statistical validity—focusing on the “low bone mass spectrum” (osteopenia + OP) avoids diluting predictive signals from the normal BMD subgroup and ensures sufficient sample size for binary classification modeling (case: OP; control: osteopenia).

Key baseline characteristics of the three initial subgroups (normal BMD, osteopenia, OP) are summarized in **Supplementary Table 1** to illustrate the rationale for excluding participants with normal BMD.

Ethical approval was obtained from the Institutional Review Board of Shenzhen People’s Hospital (approval No.: 2024-569[S]-01) for this retrospective human data analysis, and informed consent was waived due to the anonymized retrospective nature of the study.

### 2.2 Clinical variables and definitions

Clinical and laboratory data were extracted from structured electronic medical records encompassing three main categories: (1) Demographic and anthropometric characteristics: age, sex, height, weight, and systolic/diastolic blood pressure; (2) Laboratory parameters: hematologic indices (platelet count, hemoglobin concentration, mean corpuscular volume [MCV], mean corpuscular hemoglobin [MCH], metabolic profiles (total cholesterol [TC], triglycerides [TG], high-density lipoprotein cholesterol [HDL-C], fasting glucose [GLU], glycated hemoglobin [HbA1c], serum uric acid [UA]), hepatic markers (total bilirubin, alkaline phosphatase [ALP]), and inflammatory cell ratios (lymphocyte, neutrophil, basophil, eosinophil counts); (3) Calculated metabolic-inflammatory indices: anthropometric derivatives (body mass index[BMI], waist-to-height ratio [WHtR]), combined metabolic indices (triglyceride-glucose [TyG] index and its derivatives: TyG-BMI, TyG-waist circumference, TyG-WHtR), and hematologic-inflammatory markers (systemic inflammation index, lymphocyte-to-HDL-C ratio). Detailed definitions and measurement methods for all variables are provided in **Supplementary Table 2**.

Prediabetes was defined as HbA1c levels of 5.7%–6.4% (39–47 mmol/mol), in line with American Diabetes Association (ADA) criteria. Nonalcoholic fatty liver disease (NAFLD) diagnosis followed American Association for the Study of Liver Disease (AASLD) guidelines, requiring either biochemical or imaging evidence while excluding significant alcohol consumption (>14 standard drinks/week for women, >21 for men) and viral hepatitis. OP diagnosis was established by DXA (Hologic Discovery A scanner) with BMD measurements at three anatomical sites: lumbar spine (L2-L4), femoral neck, and total hip. Diagnostic classification adhered to WHO criteria: for participants aged ≥50 years, OP was defined as T-score ≤-2.5, osteopenia as T-score between -2.5 and -1.0, and normal BMD as T-score >-1.0. For individuals <50 years, ethnicity-specific Z-scores were used, with OP defined by an International Society for Clinical Densitometry (ISCD) threshold of Z-score ≤-2.0.

Study inclusion targeted adults aged ≥18 years with either OP (L2-L4 BMD T-score ≤-2.5) or osteopenia (L2-L4 BMD T-score between -2.5 and -1.0), ensuring consistent diagnostic thresholds across the cohort.

### 2.3 Feature selection

Feature selection was conducted via recursive feature elimination (RFE), an iterative ML algorithm that sequentially removes the least predictive variables to identify an optimal feature subset, balancing model accuracy, stability, and parsimony (14, 15). To enhance robustness, avoid overfitting and prevent data leakage, RFE was performed within each fold of the 10×10-fold cross-validation. Model performance was evaluated throughout the RFE process using this cross-validation approach, with the area under the receiver operating characteristic curve (AUC) as the primary performance metric.

### 2.4 Model development and internal validation

Eleven ML algorithms were implemented for predictive modeling: logistic regression (LR), random forest (RF), support vector machine with recursive feature elimination (SVM-RFE), extreme gradient boosting (XGBoost), gradient boosting decision tree (GBDT), decision tree (DT), multilayer perceptron (MLP), linear discriminant analysis (LDA), adaptive boosting (AdaBoost), Gaussian naive Bayes (GaussianNB), and light gradient boosting machine (LightGBM). All algorithms were evaluated for their ability to discriminate OP from osteopenia in individuals with low bone mass. To enhance reliability and minimize random sampling bias, 10×10-fold cross-validation was employed for all model development and evaluation procedures. Hyperparameter tuning was performed via grid search during cross-validation to identify optimal parameters for each algorithm. Final model optimization involved retraining each algorithm on the full training set using the optimal feature subset (identified via RFE) and the best-performing hyperparameters.

To evaluate the performance of the optimal LDA model under class imbalance, three strategies were used: raw data, class weighting, and SMOTE resampling. All analyses were performed using the mlr3pipelines package. The minority class weight was calculated based on sample size ratios, and SMOTE was implemented with 5 nearest neighbors. After 10 random 9:1splits, the mean and standard deviation of 8 performance metrics were calculated on the validation set. LDA assumptions were additionally assessed using the Shapiro–Wilk test for univariate normality, Henze–Zirkler test for multivariate normality, and Box’s M test for covariance homogeneity, with Q-Q plots used for visual inspection. These evaluations were performed on the final predictors retained in the LDA model.

All ML models were comprehensively evaluated using receiver operating characteristic (ROC) curve analysis, with the area under the ROC curve (AUC) as the primary discriminative metric (AUC range: 0.6–1.0). Model performance was systematically assessed using eight metrics. Primary discriminative metrics included AUC, sensitivity, specificity, and F1-score, which are robust to class imbalance. Secondary metrics included accuracy, PPV, NPV, and Brier score. The optimal prediction model was selected based on balanced performance across primary metrics, prioritizing models with minimal overfitting and clinical interpretability. To benchmark clinical applicability, the final LDA model was compared with the Osteoporosis Self-assessment Tool for Asians (OSTA) in an independent validation set (30% hold-out split from the full dataset). The optimal prediction model was selected based on comprehensive performance across all metrics in both training and validation sets—prioritizing models with balanced performance, minimal overfitting, and clinical interpretability. Key predictive features were identified based on the optimal model’s feature importance ranking.

### 2.5 Sensitivity and subgroup analyses

To compare the discriminative performance of LDA and logistic regression, predicted probabilities from 10 random splits were used. The pROC package was employed to conduct fold-wise and overall DeLong tests, and paired ROC curves with mean AUC values were plotted. We further examined multicollinearity among features, analyzed the coefficient reversal of Height, and assessed model performance after removing this variable. Variance inflation factors (VIF > 5 indicated collinearity, VIF > 10 indicated severe collinearity) were calculated using the car package.

To enhance clinical utility, the optimal classification threshold was determined by balancing the clinical consequences of false-positive and false-negative results. A false-positive outcome may lead to unnecessary confirmatory DXA scans and overtreatment, while a false-negative result risks missing individuals with OP and delaying fracture-preventive interventions. Thus, we selected the threshold that minimized the combined clinical risk of misclassification while maintaining robust discriminative performance.

Model performance was evaluated by gender subgroups. Data were stratified into overall, male, and female groups. Each group underwent 10 random 9:1 splits with logistic regression, and AUC, sensitivity, and specificity were calculated on validation sets. Bootstrap resampling (2000 iterations) was used to compute 95% CIs for male and female AUCs; significance was determined by CI overlap. The pROC package was applied for AUC and CI estimation (DeLong test was not applicable due to independent samples).

### 2.6 Model interpretability (SHAP analysis), nomogram construction and clinical utility evaluation

Model interpretability was analyzed using the optimal model via SHAP to quantify the specific contribution of each key feature to OP prediction. Feature importance analysis was performed to validate the rationality of model decisions and identify the most influential predictors (16). A points-based nomogram was constructed based on the optimal model using the ‘rms’ package (v6.5.0) in R (v4.2.2), followed by rigorous validation:(1) clinical utility evaluation via decision curve analysis (DCA), which compares net benefits across different risk thresholds; (2) calibration assessment with bootstrap-corrected curves. Analyses complied with TRIPOD-AI standards for predictive modeling transparency.

### 2.7 Statistical methods

Baseline characteristics were analyzed using the tableone package (v 0.13.2) (17). Categorical variables are presented as percentages (%) and compared using weighted χ² tests. Continuous variables were tested for normality via the Shapiro-Wilk test: normally distributed variables are reported as mean ± standard deviation (SD) and compared using Student’s t-test; non-normally distributed variables were compared using the Wilcoxon rank-sum test. Statistical significance was defined as p < 0.05.

**Figure 1:**
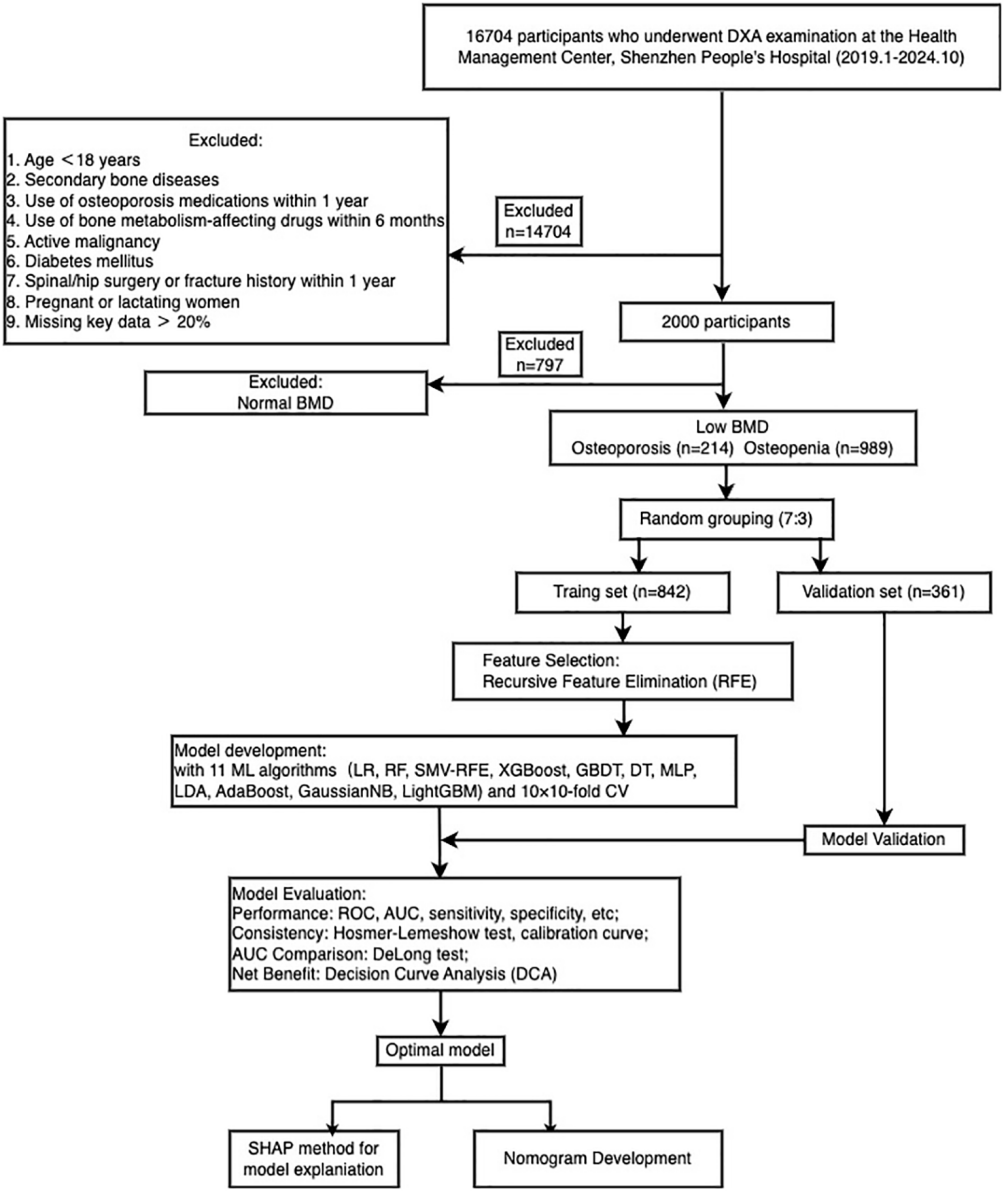
Flow chart of this study design. All abbreviations used in this figure are defined in **Supplementary Table 2**.

Significant variables (p < 0.05) from baseline analyses were selected for subsequent modeling. All ML models were developed using R 4.2.2 with the caret package (v6.0.94), employing the train() function with algorithm-specific parameters. Model discrimination was evaluated using ROC curves, with AUC and bias-corrected 95% CIs calculated from 1000 bootstrap replicates. Nomogram construction was performed using the rms package (v6.5.0). Model calibration was assessed via calibration curves (rms package) and the Hosmer-Lemeshow (HL) test (p > 0.05 indicating good calibration). Clinical utility was evaluated using DCA implemented with the ggDCA package (v1.2). A study flow chart is presented in **Figure 1**.

## Results

### Participant characteristics and feature selection

Following the application of inclusion and exclusion criteria, 2000 participants were categorized by baseline BMD into normal, osteopenic, or osteoporotic subgroups. Key characteristics differed significantly across groups (**Supplementary Table 1**).

The final analytical cohort included 1203 participants with low bone mass (osteopenia: n=989; OP: n=214). Comparative analysis revealed statistically significant differences (p < 0.05) in 17 clinical variables between the osteopenia and OP groups (**Table 1**). Participants with OP were generally older, had a higher proportion of females, and exhibited distinct profiles in anthropometric, metabolic, and hematologic parameters. These differential variables were selected as candidate predictors for model development.

**Table 1:** Comparison of baseline characteristics between osteopenia and osteoporosis groups.

Variable	Level	Overall	Osteopenia	OP	p
n		1203	989	214	
Age		51.57 (10.57)	50.43 (10.12)	56.85 (11.04)	<0.001
Gender (%)	Female	503 (41.8)	386 (39.0)	117 (54.7)	<0.001
	Male	700 (58.2)	603 (61.0)	97 (45.3)	
Height		1.65 (0.08)	1.65 (0.08)	1.61 (0.08)	<0.001
Weight		64.42 (10.64)	65.212 (10.62)	60.72 (9.96)	<0.001
SBP		120.42 (17.01)	119.39 (16.56)	125.22 (18.25)	<0.001
DBP		72.57 (11.25)	72.30 (11.27)	73.83 (11.09)	0.072
PLT		245.80 (56.58)	247.23 (56.42)	239.18 (56.97)	0.059
HGB		141.82 (15.34)	142.04 (15.67)	140.79 (13.68)	0.277
PLT_width		11.94 (2.35)	11.95 (2.32)	11.86 (2.47)	0.618
PLT_volume		10.03 (0.86)	10.02 (0.85)	10.07 (0.93)	0.491
TC		5.25 (1.04)	5.21 (1.02)	5.42 (1.11)	0.006
TG		1.52 (1.02)	1.53 (1.03)	1.50 (0.98)	0.675
HDL-C		1.44 (0.35)	1.42 (0.35)	1.51 (0.37)	0.002
TBIL		15.05 (6.42)	15.18 (6.69)	14.49 (4.96)	0.158
GLU		5.21 (1.20)	5.15 (1.09)	5.51 (1.59)	<0.001
HBA1c		5.78 (0.68)	5.74 (0.64)	5.97 (0.81)	<0.001
ALP		75.19 (23.30)	73.52 (23.04)	82.86 (22.99)	<0.001
LR		34.12 (7.73)	34.10 (7.66)	34.20 (8.08)	0.862
ALC		2.00 (0.59)	2.00 (0.59)	2.02 (0.59)	0.584
UA		374.69 (92.53)	379.64 (93.60)	351.81 (83.90)	<0.001
MCHC		326.87 (11.09)	326.88 (11.40)	326.84 (9.55)	0.964
MCH		29.72 (2.45)	29.64 (2.52)	30.09 (2.01)	0.015
MCV		90.87 (6.10)	90.61 (6.25)	92.05 (5.22)	0.002
NLR		55.58 (8.13)	55.58 (8.09)	55.59 (8.34)	0.994
BLR		0.65 (0.31)	0.65 (0.31)	0.64 (0.27)	0.498
ELR		2.66 (2.03)	2.66 (2.03)	2.64 (2.06)	0.864
BMI		23.68 (2.91)	23.76 (2.88)	23.31 (3.00)	0.038
LHR		1.50 (0.64)	1.51 (0.64)	1.45 (0.64)	0.254
SII		13698.51 (3951.25)	13776.43 (3952.13)	13338.41 (3936.38)	0.142
TyG		1.21 (0.60)	1.20 (0.59)	1.24 (0.62)	0.34
TyG_WC		1.00 (0.55)	1.00 (0.55)	1.02 (0.55)	0.668
WHtR		0.49 (0.05)	0.49 (0.05)	0.50 (0.06)	0.047
TyG_WHtR		0.61 (0.33)	0.60 (0.33)	0.63 (0.34)	0.242
TyG_BMI		29.22 (16.03)	29.15 (15.99)	29.52 (16.28)	0.762
pre_diabetes (%)	NO	1087 (90.4)	909 (91.9)	178 (83.2)	<0.001
	YES	116 (9.6)	80 (8.1)	36 (16.8)	
NAFLD (%)	NO	795 (66.1)	639 (64.6)	156 (72.9)	0.025
	YES	408 (33.9)	350 (35.4)	58 (27.1)	

Data are presented as mean (standard deviation) for continuous variables and n (%) for categorical variables. All abbreviations used in this table are defined in **Supplementary Table 2**.

### ML model development and evaluation

**Figure 2:**
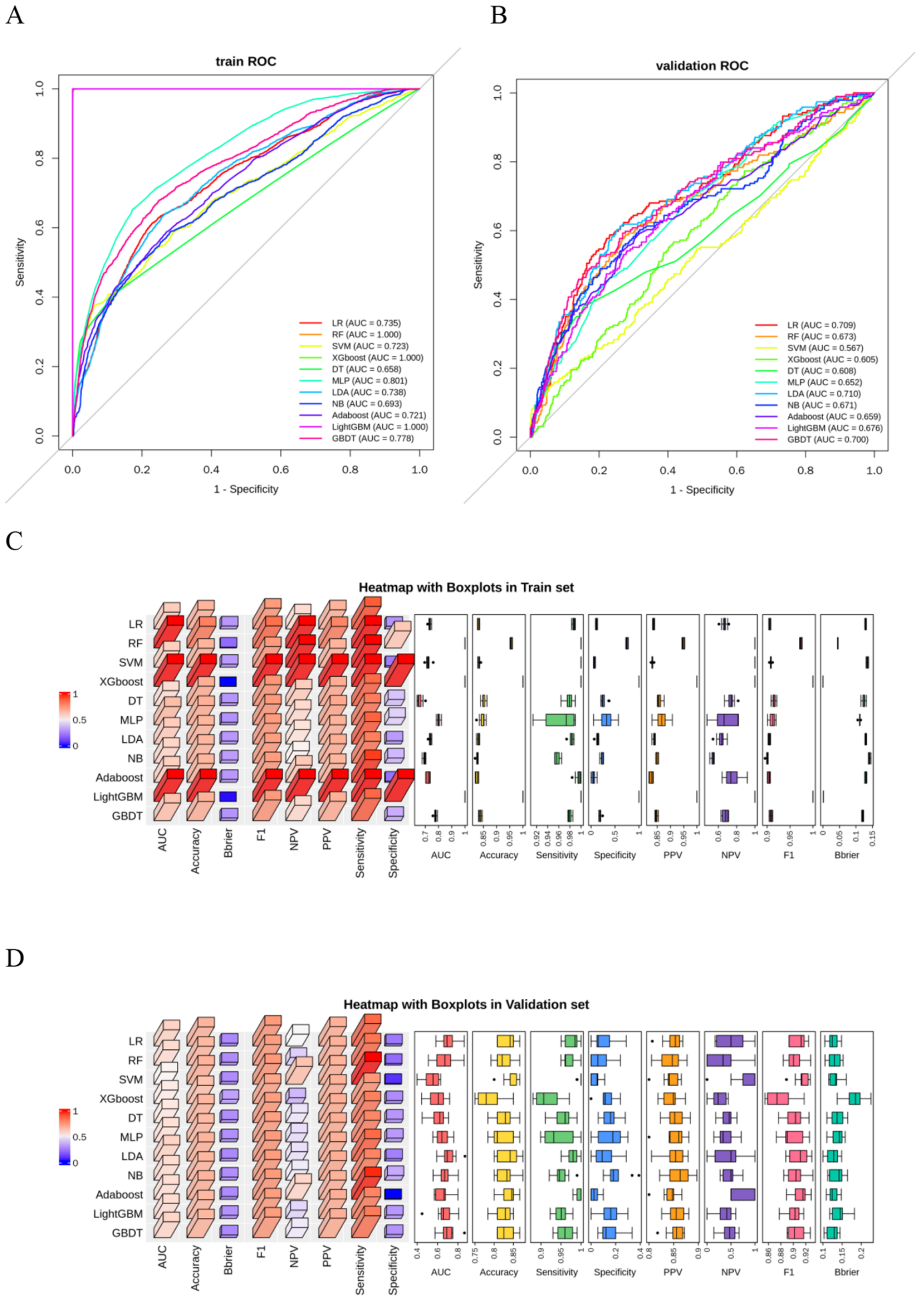
The performance of machine learning (ML) models for discriminating osteoporosis from osteopenia was evaluated in low bone mass participants using both training and validation sets. ROC curve analysis **(A, B)**.The relative performance of all models across eight key metrics was visualized via heatmap, and the distribution of these metrics was further illustrated using box plots **(C,D)**. LR, logistic regression; RF, random forest; SVM-RFE, support vector machine with recursive feature elimination; XGBoost, extreme gradient boosting; GBDT, gradient boosting decision tree; DT, decision tree; MLP, multilayer perceptron; LDA, linear discriminant analysis; AdaBoost, adaptive boosting; GaussianNB, Gaussian naive Bayes; LightGBM, light gradient boosting machine.

Given the imbalanced class distribution in the final cohort (osteopenia: n = 989; OP: n = 214), model performance was primarily evaluated using metrics less dependent on prevalence, including AUC, sensitivity, specificity, and F1-score. Accuracy was reported for completeness but was not emphasized due to its susceptibility to inflation in imbalanced datasets. Based on this evaluation framework, we systematically compared 11 ML models for discriminating OP from osteopenia. Model performance was evaluated using 10×10-fold cross-validation. The ROC curves for all models are presented in **Figures 2A, B**. Comparative heatmap and box plots summarizing performance across multiple evaluation metrics are provided in **Figures 2C, D**.

Among the models tested, complex algorithms such as XGBoost showed strong discriminative ability on the training dataset but exhibited signs of overfitting, with notable performance reduction in the validation set. In contrast, LDA model demonstrated the most balanced and stable performance. In the derivation cohort, LDA achieved an AUC of 0.738 (95% CI: 0.736–0.741) and accuracy of 0.831 (95% CI: 0.829–0.832). In the validation set, the corresponding values were an AUC of 0.710 (95% CI: 0.686–0.734) and an accuracy of 0.837 (95% CI: 0.828–0.845). The minimal decline in AUC (ΔAUC = 0.028) indicates strong generalizability, contrasting with other models that showed greater overfitting. LDA also maintained high sensitivity (0.977) and specificity, supported by consistent Brier scores and reliable calibration. Clinically, the optimal cut-off value was chosen after carefully weighing the risks of false positivity and false negativity. False positives could trigger redundant DXA referrals, whereas false negatives might fail to identify patients requiring early intervention. This clinically informed threshold ensures the model balances screening accuracy and real-world clinical utility.

**Figure 3:**
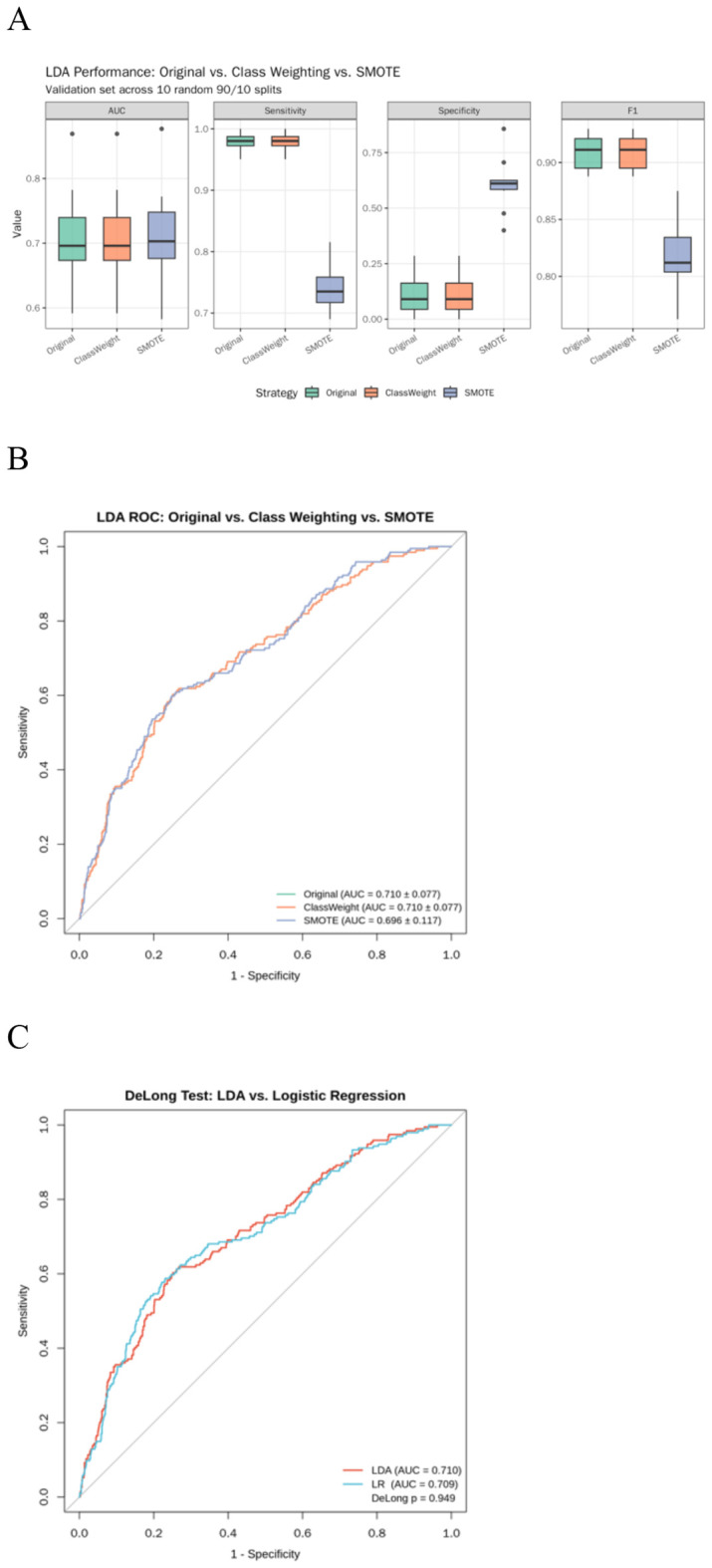
Performance of LDA under different class imbalance strategies and comparison with LR. **(A)** Box plots showing the distribution of four key evaluation metrics (AUC, sensitivity, specificity, and F1-score) for LDA across 10 random 90/10 train-validation splits. **(B)** Receiver operating characteristic (ROC) curves of LDA under the three imbalance correction strategies, with corresponding mean AUC values (± standard deviation) reported in the legend. **(C)** DeLong test comparing the ROC curves of LDA and LR on the validation set.

Furthermore, we compared the performance of LDA under three imbalance processing strategies, namely original data, class weighting and SMOTE. The original model exhibited obvious imbalance bias, with a sensitivity of 0.977 and a specificity of only 0.108. The accuracy of 0.837 was highly misleading, which was consistent with the comments from reviewers. SMOTE resampling effectively improved class balance, with the specificity increased to 0.609 and the accuracy decreased to 0.720, while the AUC remained stable at 0.712 versus 0.710 (**Figures 3A, B**). These findings highlighted the superiority of AUC as a core evaluation metric. AUC reflected the overall ranking ability of models across all thresholds and was unaffected by class distribution and decision threshold shifts. By contrast, indicators including accuracy and sensitivity fluctuated greatly with different imbalance correction approaches, which essentially indicated the movement of decision boundaries rather than the actual discriminative capacity of models. The highly consistent AUC values obtained under all three strategies verified that LDA possessed stable and reliable inherent discriminative power for distinguishing OP and osteopenia.

To formally compare the discriminative performance of LDA and LR, we conducted DeLong tests on the validation set. No statistically significant difference was observed between the two models (LDA AUC = 0.710 ± 0.077, LR AUC = 0.709 ± 0.077, Z = 0.064, p = 0.949). Consistently, all DeLong tests across the 10-fold cross-validation yielded p-values > 0.05 (range: 0.094–0.825), confirming equivalent discriminative ability (**Figure 3C**). To further evaluate LDA applicability, its assumptions were assessed using final model predictors. Univariate normality (Shapiro-Wilk), multivariate normality (Henze-Zirkler), and covariance homogeneity (Box’s M) tests indicated partial violations of LDA assumptions (**Supplementary Table 3**, **Supplementary Figure 1A**). Therefore, LDA results were interpreted cautiously, and robustness was primarily assessed via cross-validation, ROC analysis, calibration, and comparison with alternative models. Besides, to evaluate clinical relevance, LDA performance was compared with the Osteoporosis Self-assessment Tool for Asians (OSTA) (**Supplementary Table 4**, **Supplementary Figures 1B, C**). OSTA achieved an AUC of 0.691 (95% CI: 0.650–0.733), accuracy of 0.669, sensitivity of 0.645, and specificity of 0.674. In contrast, LDA showed superior performance, with an AUC of 0.736 (95% CI: 0.699–0.774), accuracy of 0.724, sensitivity of 0.650, and specificity of 0.740, indicating improved risk stratification over the conventional tool.

Although the two models exhibited comparable predictive performance, LDA was ultimately selected as the clinical prediction model in this study for the following reasons. First, the LDA model featured a concise and stable structure with high computational efficiency, which made it more suitable for primary medical institutions with limited computing resources. Second, the discriminant rules of LDA near category boundaries possessed clear linear geometric interpretations, and combined with SHAP analysis, the model could provide globally consistent directions of feature contributions. Third, although some deviations from LDA assumptions were observed, the model demonstrated stable discrimination through repeated validation and showed comparable performance with LR. Furthermore, empirical comparisons revealed that LDA slightly outperformed LR (AUC = 0.710 versus 0.709). Additionally, the prior multi-algorithm comparison was performed exclusively to verify the stability of the selected clinical features. The high consistency in predictive performance observed between LDA and LR provides indirect evidence that the predictive features included in this study exhibit favorable reliability.

### Model interpretation and clinical application

**Figure 4:**
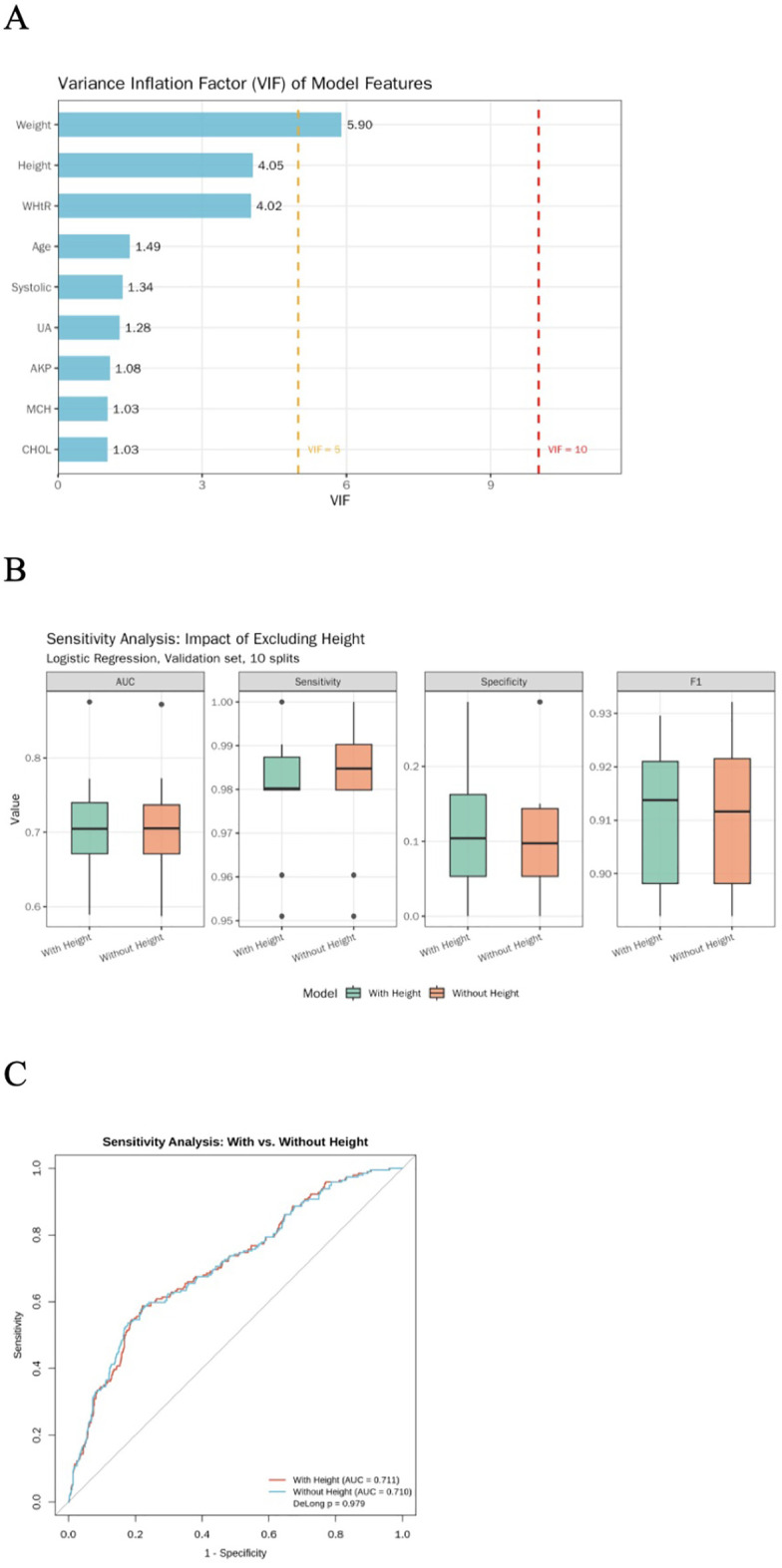
Multicollinearity assessment and sensitivity analysis of height in the logistic regression model. **(A)** VIF analysis of all predictors. **(B)** Performance comparison of models with and without height inclusion, based on 10 random train-validation splits. **(C)** ROC curves of models with and without height.

Multicollinearity assessment via variance inflation factor (VIF) analysis revealed moderate collinearity among anthropometric variables: weight (VIF = 5.90), height (VIF = 4.06), and WHtR (VIF = 4.02), while all other predictors had VIF < 1.5, with no severe collinearity (all <10) (**Figure 4A**). Pearson correlation confirmed a moderate-strong correlation between height and weight (r=0.666) (**Supplementary Table 5**). In univariate logistic regression, height showed a protective effect (coefficient = −0.524, OR = 0.592), consistent with skeletal biology. However, in the multivariable model, its coefficient reversed to +0.087 (OR = 1.091), a classic Simpson’s paradox driven by collinearity with weight. To assess the impact of height on model performance, we performed a sensitivity analysis excluding height. The results showed nearly identical distributions of AUC, sensitivity, specificity, and F1 between models with and without height (**Figure 4B**), indicating that height contributed minimally to the overall discriminative ability. Sensitivity analysis excluding height showed nearly identical performance (AUC = 0.711 vs 0.710, DeLong p=0.979) (**Figure 4C**), indicating height contributed minimally to overall discrimination and did not affect clinical utility.

**Figure 5:**
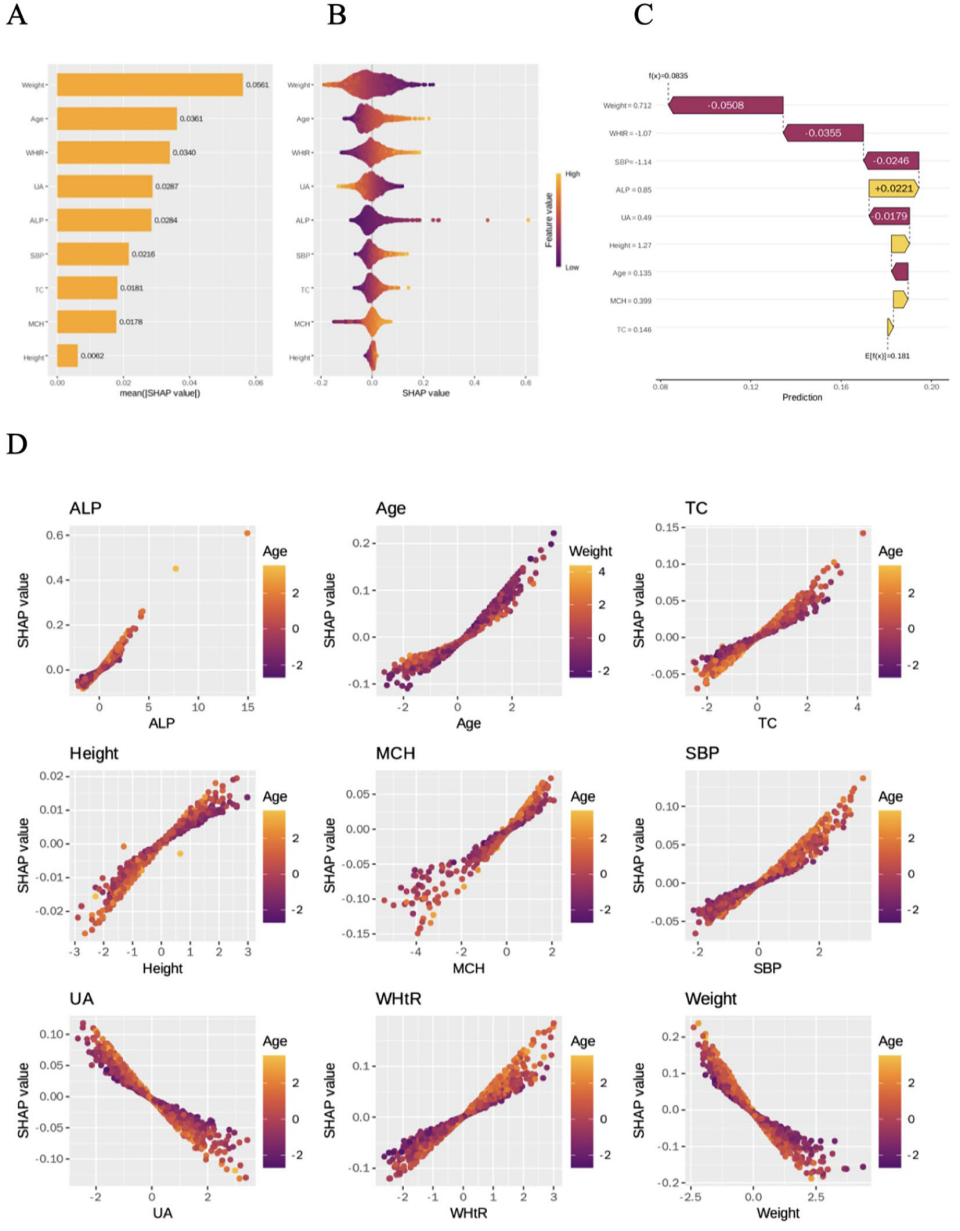
Model explanation by the SHapley Additive exPlanation (SHAP) method. **(A)** SHAP summary bar plot. This plot evaluates the contribution of each feature to the model using mean SHAP values, displayed in descending order. **(B)** SHAP summary dot plot. The probability of developing osteoporosis increases with the SHAP values of the features. Each dot represents a patient’s SHAP value for a given feature, with orange indicating higher feature values and purple indicating lower values. Dots are stacked vertically to show density. **(C)** SHAP waterfall plot. It decomposes the model’s prediction for an individual case. For this patient, weight was the most impactful negative predictor, whereas ALP was the most impactful positive predictor; WHtR, SBP, UA, and age had relatively small negative effects, while height, MCH, and TC had minor positive effects. **(D)** SHAP dependence plot. Each dependence plot shows how a single feature affects the model’s output, with each point representing a patient.

**Figure 6:**
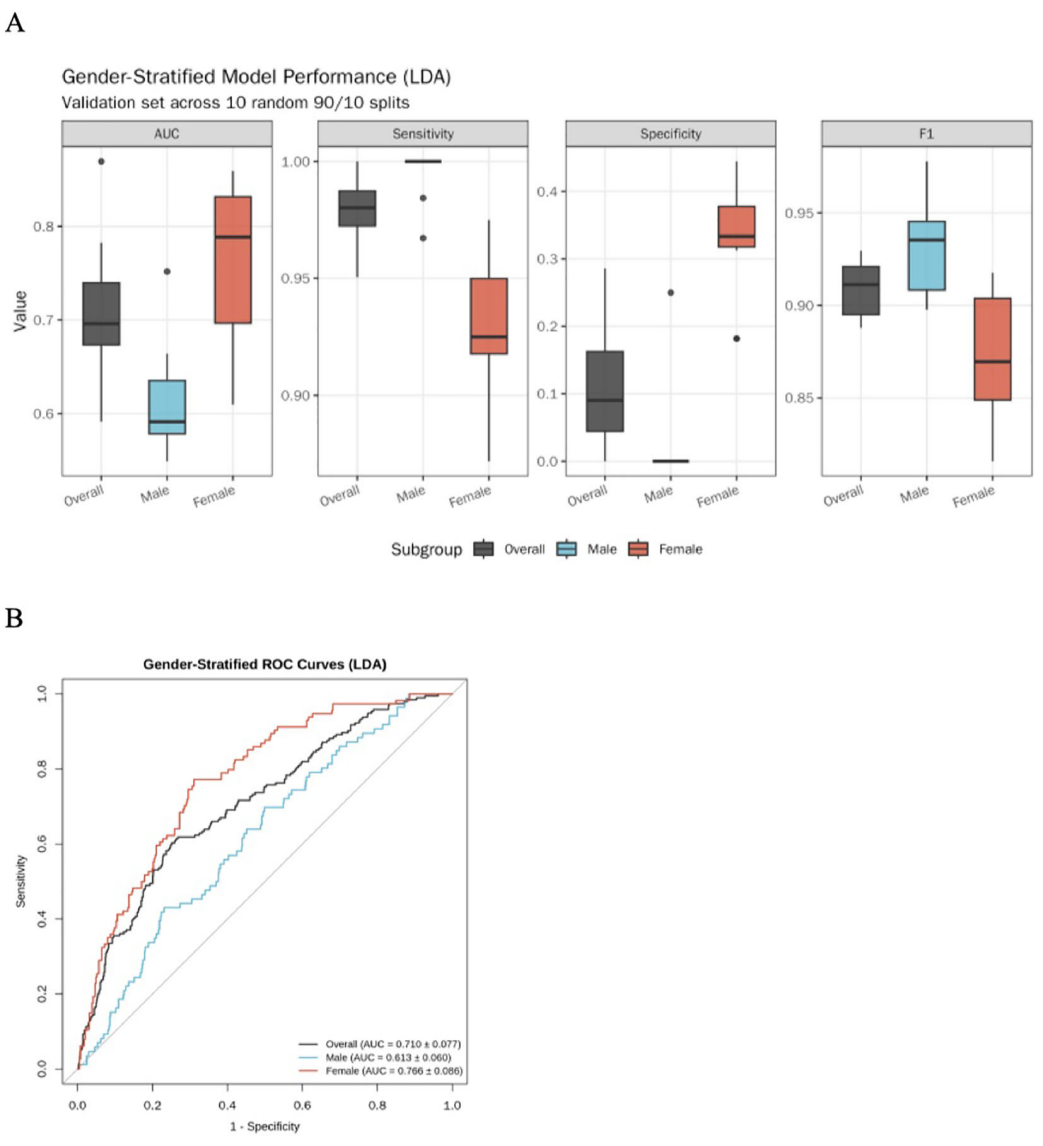
Gender-stratified performance of the LDA model for osteoporosis risk prediction. **(A)** Boxplots of key performance metrics across 10 random 90/10 train-validation splits, stratified by subgroup (Overall, Male, Female). **(B)** ROC curves for the three subgroups, evaluated on the validation set.

We performed SHAP analysis to interpret the LDA model, identifying nine key predictors of OP risk: weight, age, WHtR, UA, ALP, SBP, TC, MCH and height. Among these, weight had the strongest influence on the model’s output, followed by age and WHtR (**Figures 5A, B**). A SHAP waterfall plot illustrating the contribution of each feature for an individual patient is presented in **Figure 5C**. In this representative case, weight was the most influential negative predictor, whereas ALP contributed most positively to the risk score. WHtR, SBP, UA, and age showed modest negative effects, while height, MCH, and TC had minor positive contributions. **Figure 5D** displays the distribution of actual feature values against their corresponding SHAP values, indicating that higher age, WHtR, ALP, SBP, and TC were generally associated with elevated OP risk, whereas greater weight and higher UA levels were linked to reduced risk. To further evaluate the model’s generalizability, we performed gender-stratified subgroup analysis, dividing the cohort into male (n=700, OP = 97) and female (n=503, OP = 117) groups (**Figure 6A**). The model showed significantly better discriminative performance in females (validation 0.766 ± 0.086, 95% CI: 0.73–0.81) compared to males (male AUC = 0.613 ± 0.060, 95% CI: 0.56–0.68), with non-overlapping 95% confidence intervals indicating a statistically meaningful difference (**Figure 6B**).

**Figure 7:**
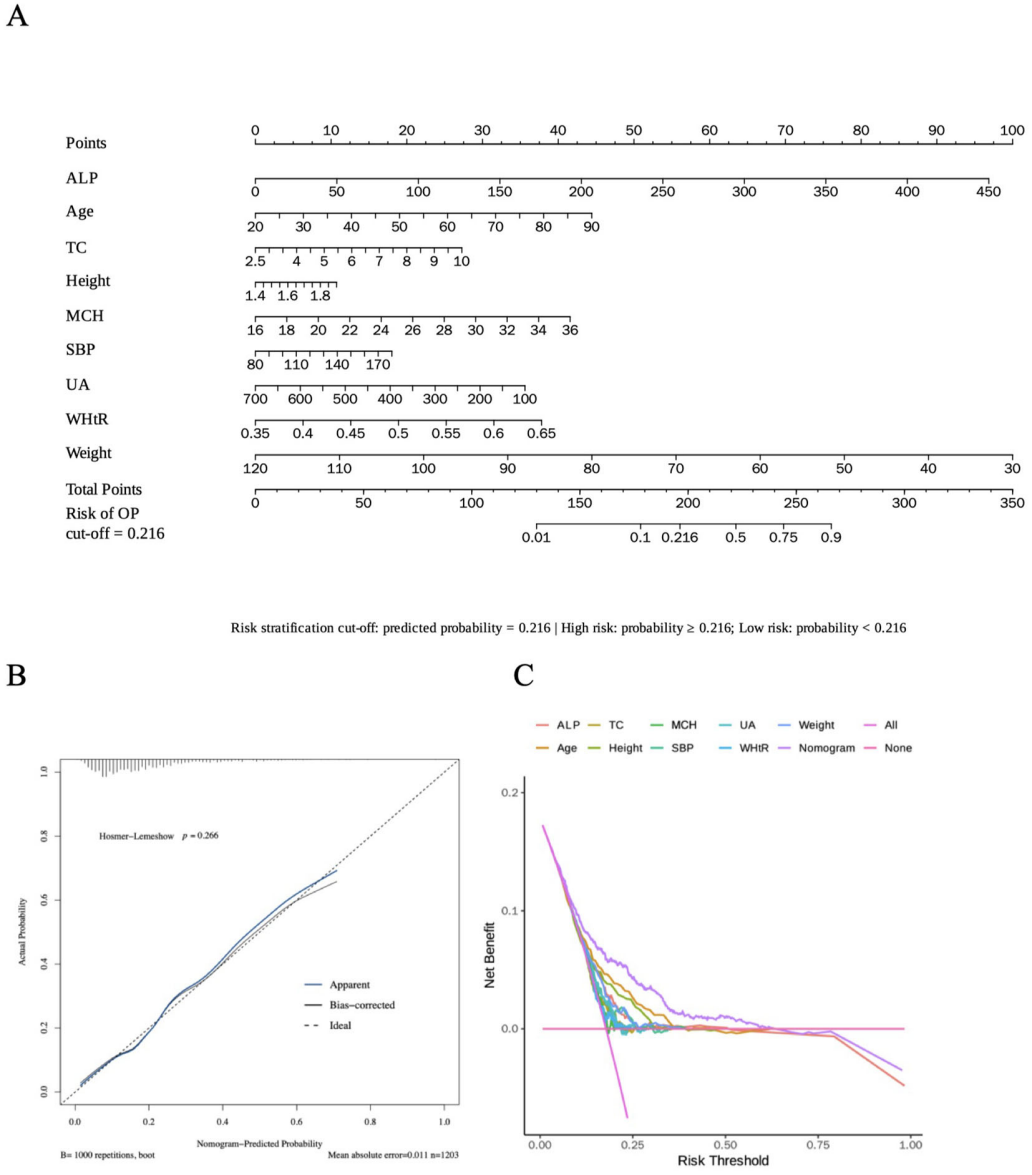
Nomogram performance and clinical utility for osteoporosis discrimination. **(A)** Forest plot showing relative importance of key predictive features. Longer bars indicate greater contribution of variables (age, weight, WHtR, UA, ALP, etc.) to the nomogram. **(B)** Calibration curve of the nomogram (n=1203). X-axis: predicted probability of OP risk; y-axis: actual observed probability. Apparent, bias-corrected (1000 bootstrap repetitions), and ideal curves are shown. Hosmer-Lemeshow test p=0.266, mean absolute error=0.011, indicating excellent calibration. **(C)** Decision curve analysis (DCA) of the nomogram. X-axis: risk threshold; y-axis: net benefit. The nomogram shows superior net benefit across most risk thresholds. ALP, alkaline phosphatase; UA, serum uric acid; WHtR, waist-to-height ratio; TC, total cholesterol; MCH, mean corpuscular hemoglobin; SBP, systolic blood pressure; OP, osteoporosis.

To support clinical translation, a nomogram was developed incorporating these predictors (**Figure 7A**). The model assigns points proportionally to risk-increasing factors, including lower weight, elevated WHtR, lower UA, higher SBP, elevated MCH, greater height, increased TC, older age, and higher ALP. The total point score corresponds to an estimated probability of OP. Calibration analysis showed close agreement between predicted and observed outcomes, with a calibration curve slope near the ideal line (**Figure 7B**). The H test yielded a p-value of 0.266, indicating no significant deviation between predicted probabilities and actual risk. Decision curve analysis (**Figure 7C**) demonstrated that the nomogram provided the highest net benefit across a wide range of threshold probabilities, supporting its clinical utility over alternative approaches.

## Discussion

This study establishes the first discriminative model for distinguishing OP from osteopenia specifically developed from populations with abnormal BMD (osteopenia or OP), filling an important methodological gap in current OP screening tools.

Previous ML models, while demonstrating improved detection capabilities for abnormal bone mineral density, have predominantly utilized mixed populations combining both normal and high-risk individuals (18). This approach inevitably compromises their predictive accuracy for clinically actionable subgroups (19). Our systematic evaluation of 11 candidate models has yielded an optimized clinical prediction tool employing only routinely available variables, offering dual advantages: enhanced accuracy in detecting abnormal bone mass populations and improved feasibility for implementation in diverse clinical setting.

The transition from technical ML performance to clinical utility presents notable challenges. While ML has shown advantages over conventional statistical methods in modeling complex biomechanical interactions (20, 21), most current prediction models are developed using mixed populations (22). This approach may explain clinicians’ ongoing challenges in accurately identifying high-risk individuals with low bone mass (23), especially in the critical osteopenia phase when preventive interventions are most effective. By contrast, our model specifically addresses this issue through its exclusive focus on abnormal bone mass cases within health examination cohorts. This targeted approach, combined with the exclusive use of routine clinical variables, substantially improves the model’s applicability for broad screening programs, particularly in resource-limited settings where advanced imaging modalities remain inaccessible.

Current OP prediction models face additional limitations beyond population selection issues. The predominant dependence on specialized biological data (e.g., DXA-measured bone mineral density, serum turnover markers like PINP and β-CTX) and high-resolution imaging (24), severely restricts their implementation in routine screening programs. Electronic medical record (EMR) systems offer a more practical alternative, providing objective and accessible data that demonstrate particular utility for large-scale population screening, especially in resource-constrained environments. Moreover, systematic reviews of postmenopausal OP prediction models reveal that while many demonstrate promising performance metrics, they frequently suffer from high risk of bias and insufficient external validation (25). This generalizability concern is exacerbated by the Western derivation of most existing models (24), as evidenced by validation studies showing heterogeneous performance of tools like the OP Self-Assessment Tool for Asians (OSTA) across different populations (26). The limitations of direct application to Chinese populations are particularly notable, with Lin et al. demonstrating significant risk estimation discrepancies among elderly males in Beijing that could lead to clinical misclassification (27).

Our study employed a systematic evaluation of 11 ML algorithms, prioritizing a balance between predictive performance and clinical applicability. This approach aligns with methodological insights from prior investigations, such as the Fasa Adult Cohort Study (FACS) (28). Previous studies suggested that logistic regression (LR) demonstrated robust discriminative ability, achieving an AUC of 0.812 in type 2 diabetes mellitus (T2DM) and 0.750 in rheumatoid arthritis cohorts (29, 30). However, while Tabib, S., et al. demonstrated that support vector machines (SVM) effectively handle high-dimensional data, their dependence on intricate parameter optimization can hinder clinical adoption (28). Similarly, other studies revealed that ensemble methods (e.g., random forests and XGBoost) exhibit strong nonlinear predictive capacity, though their practical utility is often limited by reduced interpretability and susceptibility to overfitting, particularly in smaller datasets (30, 31). Deep learning architectures, despite superior feature extraction capabilities, impose substantial computational burdens and typically require large sample sizes, which complicate real-world implementation (24). These trade-offs underscore the challenge of deploying sophisticated yet opaque “black-box” models in time-sensitive clinical workflows (24, 29).

Against this backdrop, LDA emerged as the optimal choice, offering a compelling compromise between performance and practicality. Unlike complex ensemble models prone to overfitting (e.g., XGBoost), LDA maintains stable accuracy across external validations, suggesting superior generalizability (28). LDA also provides strong mathematical transparency. By explicitly maximizing between-class variance while minimizing within-class variance, the model offers clinicians clear, direct insight into predictor contributions, eliminating the need for additional interpretation tools. Whereas logistic regression remains widely favored for its simplicity, LDA proves more robust in handling correlated features—a frequent challenge in clinical datasets. This clarity of mechanism fosters clinician confidence in predictions derived from variables such as age and BMI, which contrasts with deep learning models requiring *post hoc* explanation techniques (e.g., SHAP) for even basic interpretability (32). Thus, LDA presents a pragmatic solution for resource-constrained settings where computational infrastructure or specialized expertise may be lacking.

The SHAP analysis identified weight, WHtR, age, UA and ALP as core predictors. Previous studies also suggested the protective effect of higher body weight aligns with the mechanostat theory of bone adaptation to mechanical loading (31–33). Conversely, WHtR emerged as a particularly robust risk factor, consistent with the result of the study of Shao, Z., et al. (31). Visceral adipose tissue promotes bone resorption by secreting pro-inflammatory cytokines that activate the RANKL/RANK/OPG signaling pathway (34, 35). UA plays a significant role in OP risk models. The protective effects at moderate UA concentrations (4-4.99 mg/dL) primarily arise from its antioxidant capacity in neutralizing reactive oxygen species (36, 37).These biochemical properties substantiate observed clinical correlations where elevated serum UA levels corresponded with improved BMD across multiple skeletal sites in elderly populations (38). Large-scale epidemiological investigations, including NHANES data (37, 39), consistently confirm this beneficial relationship at physiological concentrations. However, the dose-response curve exhibits significant non-linearity, with potential detrimental effects emerging at higher UA concentrations. This biphasic relationship necessitates careful interpretation of UA’s therapeutic window in clinical models. ALP, as a marker of bone turnover, has been consistently identified as a core predictor for OP in multiple ML models (29, 31, 40). Because increased ALP levels often indicate a high bone turnover state, where the rate of bone resorption surpasses bone formation, leading to bone loss (41, 42).These findings are visualized in a nomogram, making the model transparent and clinically actionable (12, 43).

The final model incorporated additional predictors, including SBP, TC, height, and MCH. Both SBP and TC were positively associated with OP risk. A study by Jin et al. reported that elevated SBP is linked to higher OP prevalence among women in resource-limited settings, possibly due to menopause-related mechanisms (44). Similarly, a cross-sectional analysis by Zhao et al. identified a U-shaped relationship between the TC/HDL-C ratio and OP risk in older adults, particularly women (45). These findings align with the cardio-skeletal metabolic axis hypothesis, wherein chronic hypertension and dyslipidemia promote vascular calcification while paradoxically exacerbating bone mineral loss (46). In contrast, the association between MCH and OP remains less definitive. The observed mild positive association may reflect anemia-related disturbances in bone remodeling or could serve as an indirect marker of nutritional status (47). Height exhibited only a marginal effect, likely due to its correlation with bone structure rather than direct metabolic influence. Although these secondary predictors enhanced the model’s overall performance, their independent clinical relevance requires further prospective validation.

The main strength of our model is its practicality. It uses data from common health checks, enabling risk assessment where DXA is scarce. The nomogram provides straightforward scoring. For example, A 65-year-old with weight 58kg, height 160cm, WHtR 0.60, SBP 160 mmHg, MCH 34 pg, TC 5.5mmol/L, ALP100 U/L and UA 300 μmol/L yields 405 points (Age=45, Weight=50, Height=40, WHtR=50, UA = 45, SBP = 45, MCH = 45, TC = 40, ALP = 45), corresponding to about 70% OP probability. This simplicity facilitates use at the point of care. By including men and younger adults, our model also supports broader screening than guidelines focused solely on postmenopausal women (48, 49).

### Limitations and future directions

Several limitations should be acknowledged. First, the retrospective, single-center design may limit generalizability; external validation through prospective, multi-center cohorts is needed. Second, the cross-sectional nature of the data permits discrimination of disease status but not inference regarding progression; longitudinal studies are required to determine whether modifying identified risk factors (e.g., WHtR) directly influences fracture outcomes. Third, although RFE was used for feature selection, we did not systematically compare its performance against alternative strategies. Future work may systematically compare RFE with alternative feature−selection strategies, such as mutual information, regularization−based approaches, or embedded methods, to further clarify the optimal variable selection approach for osteoporosis identification in populations with low bone mass. Fourth, substantial multicollinearity among height, weight, and WHtR may have confounded the results, potentially explaining the counterintuitive positive association observed between height and osteoporosis risk; residual confounding cannot be fully ruled out despite statistical adjustments. Finally, the model performed suboptimally in the male subgroup, likely due to the more complex etiology of osteoporosis in men, lower disease prevalence (13.9%), and severe class imbalance. While this study aimed to develop a sex-neutral screening tool, resulting in the exclusion of sex as a standalone predictor, so future work should develop sex-specific models to improve diagnostic accuracy. To address these limitations, we plan to pursue multi-center external validation, incorporate additional routinely accessible biomarkers, and refine assessment tools to enhance screening precision.

## Conclusion

In summary, this study developed a pragmatic and interpretable LDA-based model for cross-sectional discrimination between OP and osteopenia in individuals with low bone mass. By utilizing only routine clinical variables, the model addresses the recognized limitation of screening accessibility in resource-limited healthcare settings. Its operational stability and reliance on clinically actionable predictors, such as WHtR and body weight, suggest potential utility in aiding early detection and rational resource allocation. Prospective validation and implementation studies in primary care are warranted to further assess its impact on clinical outcomes.

## Data Availability

The data analyzed in this study is subject to the following licenses/restrictions: The data sets generated and analyzed during this study are not publicly available but are available from the corresponding author on reasonable request. Requests to access these datasets should be directed to Wei Wang, wang.wei1@szhospital.com.

## Supplementary material

Supplementary Figure 1 LDA assumption diagnostics and comparison with OSTA. **(A)** Normality and covariance assumption tests for LDA predictors. Performance comparison **(B)** and ROC curves **(C)** of LDA and OSTA in the validation set (70/30 split).
