# Supplementary figures and images for: Explainable machine learning for osteoporosis detection in patients with osteopenia: model development and validation using routine clinical data from an Asian cohort
Source: Front Endocrinol (Lausanne). 2026 Jul 20;17:1857227. doi: 10.3389/fendo.2026.1857227 (PMC13429491; doi:10.3389/fendo.2026.1857227)

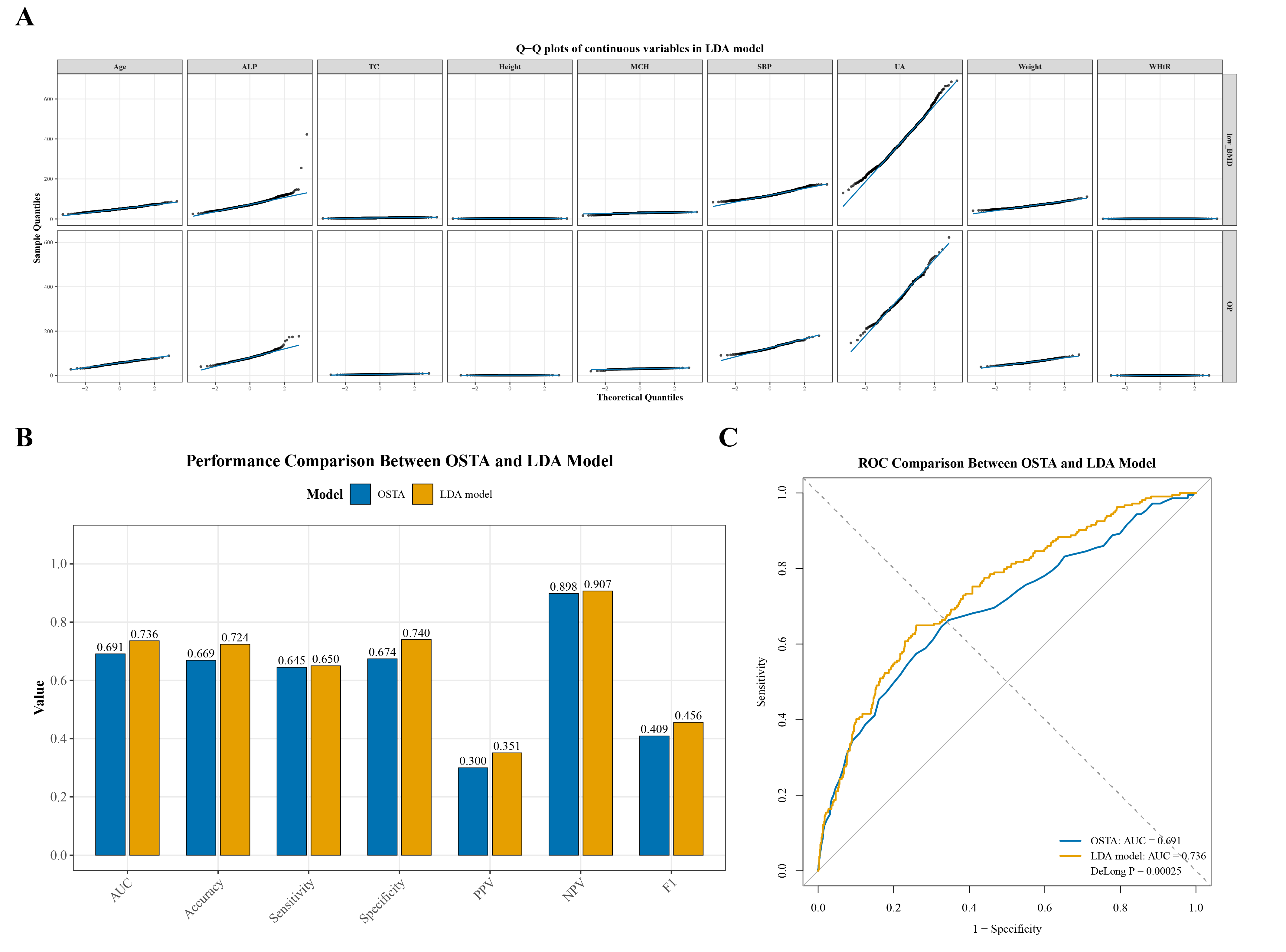

Supplement: Supplementary Figure 1 — LDA assumption diagnostics and comparison with OSTA. (A) Normality and covariance assumption tests for LDA predictors. Performance comparison (B) and ROC curves (C) of LDA and OSTA in the validation set (70/30 split). [file Image1.tif]
