# Supplementary material for: Explainable machine learning for osteoporosis detection in patients with osteopenia: model development and validation using routine clinical data from an Asian cohort
Source: Front Endocrinol (Lausanne). 2026 Jul 20;17:1857227. doi: 10.3389/fendo.2026.1857227 (PMC13429491; doi:10.3389/fendo.2026.1857227)
Supplement: Supplementary file 2 [file Table1.docx]

Supplementary Table 1. Key baseline characteristics across three bone mineral density subgroups

|  | level | Normal | Osteopenia | OP | p |
| --- | --- | --- | --- | --- | --- |
| n |  | 797 | 989 | 214 |  |
| Age |  | 47.21(8.86) | 50.43 (10.12) | 56.85 (11.04) | <0.001 |
| Gender (%) | Female | 336(42.2) | 386 (39.0) | 117 (54.7) | <0.001 |
|  | Male | 461(57.8) | 603 (61.0) | 97 (45.3) |  |
| Height |  | 1.67(0.08) | 1.65 (0.08) | 1.61 (0.08) | <0.001 |
| Weight |  | 69.51(12.01) | 65.212 (10.62) | 60.72 (9.96) | <0.001 |
| ALP |  | 66.66(17.37) | 73.52 (23.04) | 82.86 (22.99) | <0.001 |
| UA |  | 378.88(97.61) | 379.64 (93.60) | 351.81 (83.90) | <0.001 |
| BMI |  | 24.72(3.04) | 23.76 (2.88) | 23.31 (3.00) | <0.001 |
| pre_diabetes (%) | NO | 734(92.1) | 909 (91.9) | 178 (83.2) | <0.001 |
|  | YES | 63(7.9) | 80 (8.1) | 36 (16.8) |  |

Data are presented as mean (standard deviation) for continuous variables and n (%) for categorical variables. For full definitions of all abbreviations, see Supplementary Table 2.
