# Supplementary material for: Explainable machine learning for osteoporosis detection in patients with osteopenia: model development and validation using routine clinical data from an Asian cohort
Source: Front Endocrinol (Lausanne). 2026 Jul 20;17:1857227. doi: 10.3389/fendo.2026.1857227 (PMC13429491; doi:10.3389/fendo.2026.1857227)
Supplement: Supplementary file 3 [file Table2.docx]

Supplementary Table 2. Baseline statistical variables

| Variable |
| --- |
| Age |
| Gender |
| Height |
| Weight |
| Systolic Blood Pressure (SBP) |
| Diastolic Blood Pressure (DBP) |
| Platelet Count (PLT) |
| Hemoglobin Concentration (HGB) |
| Mean Platelet Distribution Width (PDW, PLT_width) |
| Mean Platelet Volume (MPV, PLT_volume) |
| Total Cholesterol (TC) |
| Triglycerides (TG) |
| High-Density Lipoprotein (HDL-C) |
| Total Bilirubin (TBIL) |
| Glucose (GLU) |
| Glycated Hemoglobin (HbA1c,) |
| Alkaline Phosphatase (ALP) |
| Lymphocyte Ratio (LR) |
| Absolute Lymphocyte Count (ALC) |
| Uric Acid (UA) |
| Mean Corpuscular Hemoglobin Concentration (MCHC) |
| Mean Corpuscular Hemoglobin (MCH) |
| Mean Corpuscular Volume (MCV) |
| neutrophils to lymphocytes Ratio (NLR) |
| Basophil Ratio (BLR) |
| Eosinophil Ratio (ELR) |
| Body Mass Index (BMI) |
| Lymphocyte to HDL Ratio (LHR) |
| Systemic Inflammation Index (SII) |
| Triglyceride-Glucose Index (TyG) |
| TyG-Waist Circumference (TyG-WC) |
| Waist-to-Height Ratio (WHtR) |
| TyG-WHtR |
| TyG-BMI |
| Prediabetes |
| Nonalcoholic Fatty Liver Disease (NAFLD) |

Note: $LHR=\frac{\text{Lymphocyte Count}}{\text{HDL}}$. $\mathrm{TyG}=\text{ln}\left( \text{TG×GLU÷2} \right)$. $\mathrm{TyG}-WC=TyG\times$WC. WHtR=$\frac{\text{WC}}{\text{Height}}$. $TyG-WHtR=\mathrm{TyG}\times WHtR. TyG-BMI=TyG\times BMI$. SII=PLT$\times$NLR.
