# Supplementary material for: Explainable machine learning for osteoporosis detection in patients with osteopenia: model development and validation using routine clinical data from an Asian cohort
Source: Front Endocrinol (Lausanne). 2026 Jul 20;17:1857227. doi: 10.3389/fendo.2026.1857227 (PMC13429491; doi:10.3389/fendo.2026.1857227)
Supplement: Supplementary file 4 [file Table3.docx]

| **Analysis** | **Group** | **Test** | **Statistic** | **P value** | **Interpretation** |
| --- | --- | --- | --- | --- | --- |
| Univariate normality | low_BMD | Shapiro-Wilk | ... | ... | Non-normal |
|  | OP | Shapiro-Wilk | ... | ... | Non-normal |
| Multivariate normality | low_BMD | Henze-Zirkler | 1.474 | <0.001 | Violation |
|  | OP | Henze-Zirkler | 1.079 | <0.001 | Violation |
| Covariance homogeneity | Overall | Box’s M | 89.375 | 0.00009 | Violation |

Supplementary Table 3. Assessment of LDA model assumptions
