# Supplementary material for: Explainable machine learning for osteoporosis detection in patients with osteopenia: model development and validation using routine clinical data from an Asian cohort
Source: Front Endocrinol (Lausanne). 2026 Jul 20;17:1857227. doi: 10.3389/fendo.2026.1857227 (PMC13429491; doi:10.3389/fendo.2026.1857227)
Supplement: Supplementary file 5 [file Table4.docx]

| Model | N | AUC (95% CI) | Cutoff | Accuracy | Sensitivity | Specificity | PPV |
| --- | --- | --- | --- | --- | --- | --- | --- |
| OSTA | 1203 | 0.691 (0.650-0.733) | -1.5 | 0.669 | 0.645 | 0.674 | 0.3 |
| LDA model | 1203 | 0.736 (0.699-0.774) | 0.196 | 0.724 | 0.650 | 0.740 | 0.351 |

Supplementary Table 4. Model Benchmarking Summary
