# Supplementary material for: Explainable machine learning for osteoporosis detection in patients with osteopenia: model development and validation using routine clinical data from an Asian cohort
Source: Front Endocrinol (Lausanne). 2026 Jul 20;17:1857227. doi: 10.3389/fendo.2026.1857227 (PMC13429491; doi:10.3389/fendo.2026.1857227)
Supplement: Supplementary file 6 [file Table5.docx]

Supplementary Table 5. Correlation analysis

|  | Height | Weight | WHtR |
| --- | --- | --- | --- |
| Height | 1 | 0.666 | -0.106 |
| Weight | 0.666 | 1 | 0.544 |
| WHtR | -0.106 | 0.544 | 1 |
